# Magnitude of postpartum depression and associated factors among women in Mizan Aman town, Bench Maji zone, Southwest Ethiopia

**DOI:** 10.1186/s12884-018-2072-y

**Published:** 2018-11-14

**Authors:** Tigistu Toru, Fantaye Chemir, Susan Anand

**Affiliations:** 1 Mizan Aman college of health science, Mizan Aman, Ethiopia; 2 School of nursing and midwifery, Jimma University, Jimma, Ethiopia

**Keywords:** Postnatal, Depression, Magnitude, Mental health, Ethiopia

## Abstract

**Background:**

The first 12 months after childbirth may represent a high-risk time for depression. In Ethiopia there is a paucity of evidence about its magnitude and associated factors during that period. So, the aim of this study was to assess the magnitude of depression and associated factors among postpartum women in Mizan Aman town, Bench Maji Zone, Southwest Ethiopia 2017.

**Methods:**

A community based cross- sectional study design was employed from March 15 to April 15, 2017. Four hundred sixty women were selected using multistage random sampling technique. Face to face interview were conducted using structured questionnaires and standardized scales. Bivariate logistic regression analysis was done to see crude association between each independent variable and outcome variable. Variables with p value < 0.25 in bivariate analysis were entered to multivariable logistic regression analysis to control for confounding. Adjusted odd ratios with 95%CI were calculated to identify independent predictors of postpartum depression.

**Result:**

Four hundred fifty-six postpartum women participated in the study giving a response rate of 99%. The magnitude of postpartum depression among the study population was 102 (22.4%, 95% CI: 19.84–24.96). Postpartum depression is relatively higher in the first 6 weeks after birth. Postpartum depression is higher among mothers with age range between 18 and 23 years (aOR 3.89 95%CI: 1.53–9.90), unplanned pregnancy (aOR 3.35 95% CI: 1.701–6.58), child having sleeping problems (aOR 3.72 95%CI: 1.79–7.72), domestic violence (aOR 2.86 95%CI 1.72–8.79), unsatisfied marital relation (aOR 2.72 95% CI 1.32–5.62), poor social support (aOR 4.30 95% CI 1.79–10.30), history of previous depression (aOR 7.38 95% CI 3.12–17.35) and substance use (aOR 5.16 95% CI 2.52–10.60).

**Conclusion:**

The magnitude of postpartum depression was high. This underlines health care planners’ needs to incorporate screening strategies for depression following childbirth.

**Electronic supplementary material:**

The online version of this article (10.1186/s12884-018-2072-y) contains supplementary material, which is available to authorized users.

## Background

Depression is a significant contributor to the global burden of disease and affects people in all communities across the world [1]. Major depressive disorder is among the top ten causes of years lived with disability (YLDs) in every country [2].

The postpartum period is well established as an increased time of risk for the development of serious mood disorders [3]. Postpartum depression can be defined as a non-psychotic depression that happens during the first 12 months after childbirth. The symptoms are no different from depression which anyone can have [4]. It is a type of depression that affects about one in 10 new mothers within the first year after childbirth and has the potential to negatively impact a new mother’s health and her ability to nurture her infant [5].

The prevalence of postpartum depression in African American women ranges from 10 to 15% [6].

A synthesis of evidence in a systematic review on common perinatal mental disorders in women in low and middle income countries revealed a pooled prevalence of postpartum common mental disorders to be higher than in high-income countries with an average prevalence rate of 19.8%. However, perinatal mental health of women living in low and middle income countries has only recently become the subject of research since the priority is given to preventing pregnancy related complications [7].

Despite the fact that postpartum depression is the major maternal mental health problem following childbirth, little was known about the magnitude of the problem and contributing factors in Ethiopia in general and it was not investigated in the study area in particular; therefore, this study was aimed to fill this gap.

## Methods

### Study area and period

This study was conducted in Mizan-Aman town from March 15 2017 to April 15 2017. Mizan Aman is Bench Maji zonal town, one of the 13th zones in the Southern Nations Nationalities and People’s Regional Government (SNNPRG). The town is located 561 Km away from Addis Ababa and 832 Km away from Hawassa (capital of SNNPRG). Mizan Aman Town is administratively structured by five kebeles and has an estimated total population of 50,113 of which 23,721 were females. Out of all females, 11,679 were women of reproductive age (15–49 yr.). The town has one teaching hospital, one health center and five health posts and all are runned by the government. On the other hand, there were nine private clinics and eight drug stores.

### Study design

Community based quantitative cross-sectional study.

### Source and study population

The source populations for the study were all postpartum women who gave birth within 12 months before data collection. Randomly selected women who gave birth within 12 months prior to data collection and who fulfilled the eligibility criteria were included. Women who gave birth within the last 12 months prior to data collection irrespective of type of delivery and outcome of delivery were included in the study.

### Sampling procedure

Sample size was determined by using single population proportion formula based on the assumptions of 19% proportion of women who developed postpartum depression from a previous study in Tigray region, Northern part of Ethiopia [8] with precision (margin of error) 5% between sample and population parameter and 95% confidence level. The final sample of 460 postpartum women was included in the study after considering finite population correction, design effect of two and 10% non-response rate.

Three among five kebeles in the town were selected using lottery method. Then, the sample was proportionally allocated to the three kebeles and simple random sampling technique was used to get 460 participants from 1720 postpartum mothers by taking the community health extension workers’ postpartum registration book as a frame.

### Measurement

An interviewer-administered structured questionnaire containing socio-demographic, obstetric, pediatric, psychosocial, substance use, psychiatric and medical illness history was adopted from previously published studies. Moreover, 9- items Patient Health Questionnaire, 3-item Kansas Marital Satisfaction Scale and 3-item Oslo Social Support Scale were used to assess the magnitude of postpartum depression, level of marital satisfaction and level of social support respectively (Additional file 1).

The questionnaire is prepared in English and then translated to local language (Amharic) as well as back to English by bilingual experts in order to check consistency. Pretest was done by taking 5% of the sample in ‘Kometa’ kebele which was not included in the actual data collection phase to assess the face validity of the questionnaire.

### Data collection

Six trained diploma nurses collected the data while three BSc nurses closely supervised data collection procedures. The principal investigator followed the overall data collection procedure plan. Face to face interviewer administered method of data collection was employed using structured questionnaires. Actual data collection was conducted after minor modification was made on tools based on pre-test findings. To assure the quality of the data, data collectors and supervisors were trained for two days on the clarity of tools and overall data collection procedures to standardize interview procedures and reduce interviewers’ bias.

### Data analysis

Data was entered to Epi- Data version 3.1 and then exported to SPSS version 21 for analysis purpose. Before analysis, missing values were checked and new categories created as needed. Descriptive statistics (frequencies, percentages) were performed. Bivariate logistic regression analysis was employed to determine the crude association between the dependent variable with each independent variable. Those exposure variables with *p*-values < 0.25 were entered into multivariable logistic regression analysis to control for confounders. Adjusted odds ratios with 95% CI were used to identify statistically significant associations between dependent and independent variables.

### Operational and term definitions

#### Postpartum depression

In this study, postpartum depression was measured by using the Patient Health Questionnaire scale with 9 items and total sum score of 27. PD was categorized in to depressed (total sum score ≥ 10) and not depressed (total sum score < 10) [9].

#### Infant sleeping problem

Infants sleeping problem was defined based on maternal response of infants sleep duration as irregular or not sufficient.

#### Baby blues

Were measured by mother’s experience of mood instability, depressed mood, sadness, irritability, anxiety, lack of concentration and/or feelings of dependency within two weeks following childbirth.

#### Level of marital satisfaction

Was assessed using Kansas Marital Satisfaction scale with 3 items and total sum score of 21. Total sum score of 17 and above was used as a cutoff point to identify women satisfied with their marital relation. Total sum score below 17 indicates dissatisfaction with marital relation [10].

#### Domestic violence

Is any behavior within an intimate relationship that causes physical, psychological or sexual harm and reported by mothers with yes or no items. Mothers were categorized as a victim of domestic violence when they experienced any of the harms (physical, psychological and sexual) in their intimate relationship.

#### Social support

Measures of available support that women perceive or believe that people in their social network would provide in times of need. Measured by 3- Items Oslo Social Support Scale with total sum score of 15 and categorized as; a score of 3–8 is poor support, 9–11 is moderate support and 12–15 is strong support [11].

#### Women substance use history

Is history of using any kind of substances such as fabricated or local alcohol, chat chewing, cigarette or tobacco smoking and other such addictive substance during pregnancy or after childbirth measured by yes or no items with at least one yes response.

#### Husband substance use

Husband’s history of using any kind of substances such as fabricated or local alcohol, chat chewing, cigarette or tobacco smoking and other such addictive substance and measured by at least one yes response.

## Result

From four hundred sixty women, 456 women completed the interview which gives a response rate of 99%.

### Socio demographic characteristics of participants

All the study participants age ranged from 18 years to 39 years with majority 192 (42.1%) between 18 and 23 years. Four hundred forty-three (97.1%) of women were married, 163 (35.7%) were from Bench Ethnic group, 179 (39.3%) participants followed Orthodox religion, and 211 (46.3%) had attended up to diploma level and above. Regarding husband’s educational level and occupation, 152 (34.3%) had diploma and above and 229 (51.7%) were merchants. One hundred fifty-three (33.6%) participants reported that their average family monthly income from all sources was less than 1000 ETB (~ 35 USD) (Table 1).

**Table 1:** Socio-demographic characteristics of postpartum women in Mizan Aman Town, Bench Maji Zone, SNNPR, Ethiopia from March 15, 2017 to April 15, 2017 (*n* = 456)

Variables	Category	Frequency	Percentage
Age	18–23	192	42.1
	24–29	167	36.6
	> = 30	97	21.3
Marital status	Married	443	97.1
	Others	13	2.9
	Others: divorced, widowed, separated		
Ethnicity	Bench	163	35.7
	Amhara	99	21.7
	Keffa	79	17.3
	Oromo	46	10.1
	Gurage	24	5.3
	Tigre	24	5.3
	Others	21	4.3
Religion	Orthodox	179	39.3
	Protestant	162	35.5
	Muslim	94	20.6
	Catholic	21	4.6
Women education	Unable to read and write	16	3.5
	Elementary	147	32.2
	Secondary	82	18.0
	Diploma and above	211	46.3
Women occupation	Government employee	199	43.6
	Housewife	166	36.4
	Merchant	61	13.4
	Private employee	25	5.5
	Student	5	1.1
Husband education	Unable to read and write	33	7.4
	Elementary	147	33.2
	Secondary	111	25.1
	Diploma and above	152	34.3
Husband occupation	Merchant	229	51.7
	Government employee	117	26.4
	Private employee	75	16.9
	Others	22	5
	Others: student, farmer, contractor, carpenter		
Average family monthly income(ETB)^a^	< 1000	153	33.6
	1000–2000	179	39.2
	> 2000	124	27.2

^a^1 USD = ~28ETB

### Magnitude of postpartum depression

**Fig. 1:**
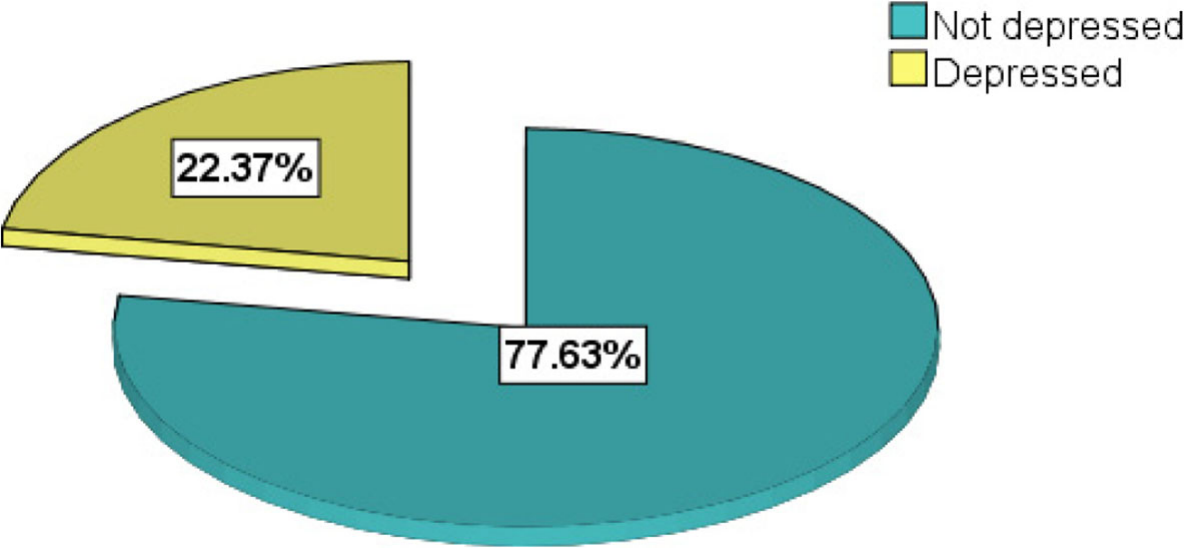
Prevalence of postpartum depression among women in Mizan Aman Town, Bench Maji Zone, Southwest Ethiopia 2017

According to this study, the maximum sum score which was recorded by participants was 16. The overall prevalence of postpartum depression among the study participant was found to be 102 (22.4%) (Fig. 1). Among those depressed, the prevalence of depression was more or less similar during the first six weeks 38 (37.3%), during 6 weeks to 6 months 33(32.4%) and above 6 months’ postpartum period 31(30.4%).

### Factors associated with postpartum depression

Among all the explanatory variables entered to bivariate binary logistic regression analysis, nineteen of them with *p*-value < 0.25 entered into multivariable analysis to control for confounding. These variables include age, marital status, women’ occupation, family monthly income, gravidity, status of recent pregnancy, complication after childbirth, difficulty of child feeding, child sleeping problem, domestic violence, marital satisfaction, social support level, previous depression, family mental illness, recent medical illness and substance use. Eight independent predictor variables for occurrence of postpartum depression were identified in the multivariable binary logistic regression model.

Accordingly, mothers prone to postpartum depression were likely to be those with; history of previous depression (aOR: 7.38, 95%CI: 3.12–17.35), substance use (aOR: 5.17, 95%CI: 2.52–10.60), poor social support (aOR: 4.30, 95%CI: 1.79–10.30), age range between 18 and 23 years (aOR: 3.89, 95%CI: 1.53–9.90), infant sleeping problems (aOR: 3.72, 95%CI: 1.79–7.72), unplanned pregnancy (aOR: 3.35, 95%CI: 1.70–6.58), domestic violence (aOR: 2.86 95% CI 1.72,8.79), and unsatisfied marital relation (aOR: 2.72, 95%CI 1.32–5.62) (Table 2).

**Table 2:** Predictors of postpartum depression in multivariable analysis among postpartum women in Mizan Aman Town, Bench Maji Zone, Southwest Ethiopia March to April, 2017

Variables	Category	Depressed *N* (%)	Not depressed N (%)	aOR(95% CI)
Age	18–23	71(37)	121(63)	3.89(1.5,9.9)^a^
	24–29	19(11.4)	148(88.6)	0.72(0.2,2.1)
	> = 30	12 (12.4)	85 (87.6)	1
Recent pregnancy status	Unplanned	71(33)	126(67)	3.35(1.7,6.6)^a^
	Planned	31(15)	228(85)	1
Child sleeping problem	Yes	54(55)	45(45)	3.72(1.8,7.7)^a^
	No	48(13)	309(87)	1
Domestic violence	Yes	40(42.5)	54(57.5)	2.86(1.7,8.8)^a^
	No	62(20)	299(80)	1
Marital satisfaction	Dissatisfied	70(36.5)	129(63.5)	2.72(1.3,5.6)^a^
	Satisfied	26(10)	221(90)	1
Social support	Poor	53(54)	41(46)	4.29(1.8,10.3)^a^
	Moderate	24(17)	137(83)	2.00(0.8,4.9)
	Good	25(12)	176(88)	1
Previous depression	Yes	40(71)	27(29)	7.38(3.1,17.4)^a^
	No	62(12)	327(88)	1
Substance use	Yes	52(49)	57(51)	5.16(2.5,10.5)^a^
	No	50(12)	297(88)	1

^a^Statistically significant association, 1 reference group

## Discussion

The magnitude of postpartum depression in this study was 22.4% (95% CI: 19.84–24.96). This was higher when compared with findings from Southern India (12%) [12], Sudan (9.2%) [13], Greenland (8.6%) [14] and Northern part of Ethiopia (19%) [8]. The variation might be due to differences in study setting (facility based in India), or measurement scale (Edinburgh depression screening tool in most of the studies). The higher postpartum depression prevalence in this study may also be explained by low living standards in the study setting where ¾ (72.8%) of the mothers had low socio-economic status. This finding was in agreement with the findings from Sudan [13] and Egypt [15] where mothers with low household income were more likely to develop PPD.

The magnitude of postpartum depression in this study was found to be lower than findings from Egypt (49.5%) [15], South Africa (50.3%) [16] and Nigeria (28%) [17]. The discrepancy might also be attributed to differences in sampling methods utilized since we used 12-month period while the comparable studies used less than 6 months which may make sample size smaller. Social desirability bias is most likely since we have employed interviewer administered method of data collection technique. This could have made them not feeling safe and therefore not willing to express their true feelings and ideas. The presence of a different measurement scale for measuring depression might also contribute to the discrepancy in the prevalence in both directions.

Postpartum depression was more common among women age between 18 and 23 years compared with thirty years and above. This might be due to younger women being more exposed to emotional distress as they experience childbirth for the first time and extra burden was added for caring infants as well as the whole family in this particular period. This finding is consistent with findings in Sudan and North America [13, 18].

Women who did not plan their pregnancy were nearly three times more likely to experience PPD when compared with their counter parts. This is in line with findings in South Africa [16] and Cameroon [19]. This might be explained as inadequate preparation for pregnancy, childbirth, and nursing, leading to mothers to feel anxious, helpless and have less (or no) ability to cope with all the changes and challenges babies bring.

Mothers whose infants had sleeping problems were nearly four times more likely to be depressed when compared with mothers whose infants sleep regularly. The findings are consistent with findings in Cameroon [19] and Egypt [15]. This could be explained as sleeping problems in the early years would cause maternal sleep loss and feelings of fatigue contributing to maternal depressive symptoms. The current study also identified that the odds of postpartum depression among women who experienced domestic violence from their partner was nearly four times higher than those who did not. This is in line with findings in Egypt [15], Sudan [13] and Japan [20]. Violence of any kind has a devastating physical, psychological, behavioral and developmental effect on the victims.

The occurrence of postpartum depression was high among women who were previously diagnosed for depression. This is explained in studies conducted in China and Canada [21, 22]. Women with unsatisfied marital relations and poor social support were more likely to experience postpartum depression. This finding was supported by findings in Cameroon [19], Pakistan, Iran [23] and South Asia [24]. Lack of support, love, affection and guidance of husband on top of a stressful childbirth event could make women vulnerable to PPD. Women who have a history of substance use were nearly five times more likely to have postpartum depression than non-users. Similarly, this is supported by findings from a systematic review [25]. Postpartum substance use could limit a mother’s ability to stay emotionally connected to her infant, adjust to his or her rhythms and behaviors, and anticipate or follow his or her development.

## Strengths and limitations of the study

The study used a community based design which helped to address those mothers who were unable to visit health care facilities enhancing generalizability of the findings. Standardized tools which were tested for their validity according to Ethiopian context were employed to diagnose depression. Women may commit social desirability bias in order to avoid discrimination that could follow after stating that they have mental health problems. The cross-sectional nature of the study limits the capacity to draw any causal implications.

## Conclusion

The magnitude of postpartum depression among women in the study was significantly higher requiring better strategies to integrate maternal mental health care packages during prenatal and postpartum periods. The study also reported different factors that contribute to the occurrence of postpartum depression such as unplanned pregnancy, presence of domestic violence, unsatisfied marital relation, poor social support and substance use history. Increasing awareness and access to contraceptive options during the preconception period will enhance maternal pregnancy planning. Health institutions should devise a strategy to create and strengthen support groups that create opportunities for positive interpersonal exchanges and emotional support for the mothers during pregnancy and after childbirth. In the community, health extension workers could provide anticipatory guidance for new mothers, such as organizing peer-support group fora and facilitating vicarious experience, as it is recognized as a parental self-efficacy enhancement strategy.

## Additional file

Additional file 1: Measurement tools for data collection. The section containing structured questionnaires and measurement scales used to assess the study variables. (DOCX 35 kb)

## Abbreviations

aOR: Adjusted odds ratio
CMD: Common mental disorders
ICD-10: International classification of diseases, 10th revision
IPV: Intimate partner violence
LICs: Low- income countries
PND: Postpartum depression
WHO: World Health Organization
