# Supplementary material for: Magnitude of postpartum depression and associated factors among women in Mizan Aman town, Bench Maji zone, Southwest Ethiopia
Source: BMC Pregnancy Childbirth. 2018 Nov 14;18:442. doi: 10.1186/s12884-018-2072-y (PMC6237000; doi:10.1186/s12884-018-2072-y)
Supplement: Supplementary file 1 — Measurement tools for data collection. The section containing structured questionnaires and measurement scales used to assess the study variables. (DOCX 35 kb) [file 12884_2018_2072_MOESM1_ESM.docx]

**File 2: English version of Questionnaire used for this study(Adapted)**

**PART 1: WOMAN’S SOCIODEMOGRAPHIC CHARACTERISTICS**

| Questions  code | Questions | Responses | skip |
| --- | --- | --- | --- |
| 101 | What is your age in completed years? | ………..Year  99. Don’t know |  |
| 102 | What is your marital status currently? | 1.married  2. single  3. Widowed  4. Divorced  5. Separated  6. Others (specify)_____________ |  |
| 103 | Which ethnic group do you belong? | 1. Bench  2. Amhara  3.Oromo  4. Kaffa  5.Gurage  6.Others (specify)-------------------- |  |
| 104 | Which religion do you follow ? | 1.Orthodox  2.Muslim  3. Catholic  4. Protestant  5.Others ___________________ |  |
| 105 | What is your educational level? | 1.unable to read and write  2. Able to read and write  3. Elementary  4. Secondary  5.preparatory  6.Diploma  7.Degree and above |  |
| 106 | What is your partner’s/husband’s educational level? | 1.unable to read and write  2. Able to read and write  3. Elementary  4. Secondary  5.preparatory  6.Diploma  7.Degree and above |  |
| 107 | What is your occupation? | 1.Merchant  2. House wife  3. Government employee  4. Private employee  5.Student  6.Others (specify__________ |  |
| 108 | What is your husband’s occupation? | 1.Merchant  2.Government employee  3. Private employee  4.Student  5.Others(specify___________ |  |
| 109 | What is the approximate monthly household income from all the sources? | ---------------Eth.birr |  |

**PART 2: OBSTETRICS FACTORS**

| 201 | \| Total number of pregnancy including the last one ? \| \| --- \| | --------- |  |
| --- | --- | --- | --- | --- |
| 202 | No_ of children | 1. ≤3 children  2. ≥4 children |  |
| 203 | What order was your last birth? | 1.First  2.second  3.Third  4.fourth and above |  |
| 204 | What was your last pregnancy status? | 1.planned  2.unplanned |  |
| 205 | Did you experience any complications during your last pregnancy? | 1.yes  2.no 207 | skip |
| 206 | If your response is yes for Q207, What type of complication did you encountered? | 1.Bleeding  2.Hypertension  3.malaria infection  4.others |  |
| 207 | Where did you give your last birth? | 1.home  2.health facility  3.other place(specify)----------- |  |
| 208 | What was the mode of your last delivery? | 1.spontanoaus vaginal  2.cesserian section  3.others(specify) |  |
| 209 | What was your last delivery outcome | 1.Alive  2.Still birth  3.Alive but dead soon  4.others |  |
| 210 | Did you experience any complication after childbirth? | 1.yes  2.no |  |
| 211 | If your response is yes for Q210, What type of complication did you encountered after giving your last birth? | 1.bleeding  2.genital Trauma  3.uncontrollled hypertension  4.infection  5.others |  |
| 212 | What was your gestational age of your last pregnancy at childbirth? | -----------weeks  --------------months  999.I don’t know |  |
| 213 | Did you experience any abortion previously? | 1.yes  2.no |  |
| 214 | If yes, how many times? | 1. once  2. twice and above |  |

**PART 3: PEDIATRICS FACTORS**

| 301 | Sex of your baby | 1.Male  2.Female |  |
| --- | --- | --- | --- |
| 302 | Desired sex of your baby | 1.Male  2.Female  3.Unspecified |  |
| 303 | What was birth weight of your child ? | ---------------gram  5.I don’t know |  |
| 304 | What did you start to feed your infant immediately after birth? | 1.Breast feeding  2.Bottle feeding  3.Other (specify------------ |  |
| 305 | If your answer is 1 for Q304,did you fed for completed 6 months? | 1.yes  2.no |  |
| 306 | Did you experience difficulty to feed your baby? | 1.yes  2.no |  |
| 307 | Is there difficulty of sleeping of your baby | 1.yes  2.no |  |
| 308 | Infant illness at any time after birth? | 1.yes  2.no |  |
| 309 | Was there history of less than one-year death? | 1.yes  2.no |  |
| 310 | Within in the last 2 weeks, did you experience symptoms like mood instability, depressed mood, sadness, irritability, anxiety, lack of concentration and/or feelings of dependency?(*tick yes if they apply to any of the signs* ) | 1.yes  2.no |  |

**PART 4: PSYCHOSOCIAL DETERMINENTS**

| 401 | Did you have recent violence from your partner/ husband? | 1.yes  2.no |  |
| --- | --- | --- | --- |
| 402 | If your response is yes for the above, which one? | 1.physical  2.psychological  3.psychological and physical  4.sexual  5.others |  |
| 403 | Was there close family member death in this year? | 1.yes  2.no |  |
| 404 | Family member sickness currently? | 1.yes  2.no |  |

**PART 5: PSYCHIATRIC AND CHRONIC PHYSICAL ILLNESS**

| 501 | history of depression? | 1.yes  2.no |  |
| --- | --- | --- | --- |
| 502 | Family history of mental illness? | 1.yes  2.no |  |
| 503 | Were you on treatment for chronic illness? | 1.yes  2.no |  |
| 504 | If yes, which type? | 1.Anemia  2.Inreased blood pressure  3.Increased glucose level  4.Tuberclosis  5.others |  |
| 505 | Were you currently using any contraceptives? | 1.yes  2.no |  |
| 506 | If yes, which one? | 1.OCPs  2.Injectable  3.Implants  4.IUCD  5.Others(specify) |  |

**PART 6: HISTORY OF SUBSTANCE USE**

| 601 | Substance use history during pregnancy or after childbirth? | 1.yes  2.no |  |
| --- | --- | --- | --- |
| 602 | If yes, which substance? | 1.ciggarrete  2.chat  3.Alcohol  4.others |  |
| 603 | Is your husband using the above substances? | 1.yes  2.no |  |
| 604 | If yes, which substance? | 1.ciggarrete  2.chat  3.Alcohol  4.others |  |

**ANNEX IV: PATIENT HEALTH QUESTIONNAIRE (PHQ-9)**

code: ___________________________

Postnatal Days------------------weeks ---------------months---------------------

Date: ___________________

1. Over the last 2 weeks, how often have you been bothered by any of the following problems?

|  | **Not at all(0)** | **Several days (1)** | **More than half the days (2)** | **Nearly every day (3)** |
| --- | --- | --- | --- | --- |
| 1. Little interest or pleasure in doing things. |  |  |  |  |
| 1. Feeling down, depressed, or hopeless |  |  |  |  |
| 1. Trouble falling/staying asleep, sleeping too much. |  |  |  |  |
| 1. Feeling tired or having little energy. |  |  |  |  |
| 1. Poor appetite or overeating. |  |  |  |  |
| 1. Feeling bad about yourself, or that you were a failure, or have let yourself or your family down |  |  |  |  |
| 1. Trouble concentrating on things, such as reading the newspaper or watching TV. |  |  |  |  |
| 1. Moving or speaking so slowly that other people could have noticed. Or the opposite; being so fidgety or restless that you have been moving around more than usual. |  |  |  |  |
| 1. Thoughts that you would be better off dead or of hurting yourself in some way. |  |  |  |  |

2. If you checked off any problem on this questionnaire so far, how difficult have these

Problems made it for you to do your work, take cwere of things at home, or get along with

Other people?

** Not difficult  somewhat  Very  extremely**

**At all difficult difficult difficult**

Annex V: Kansas Marital Satisfaction Scale

The KMSS is a short and precise measurement including three questions:

Each item on the scale has a possible score ranging from one to seven. Scores of seven indicate a high degree of satisfaction while scores of one indicate a low degree of satisfaction. A total score on the scale could range from 3 to 21.

Extremely Very Somewhat Somewhat Very Extremely

Item Dissatisfied Dissatisfied Dissatisfied Mixed Satisfied Satisfied Satisfied

1. How satisfied

were you with your 1 2 3 4 5 6 7

Marriage?

2. How satisfied 1 2 3 4 5 6 7

were you with your

husband as

a spouse?

3. How satisfied 1 2 3 4 5 6 7

were you with your

relationship with

your husband?

Annex VI- OSLO-3: SOCIAL SUPPORT SCALE

1. **Oslo 1**: How many people were you so close to that you can count on them if you have great personal problems?

**1**= none **2**= 1–2 **3**= 3–5 **4**= 5+

1. **Oslo 2**: How much interest and concern do people show in what you do?

**1**=none **2**= little **3**= uncertain **4**=some **5**= a lot

1. **Oslo 3**: How easy is it to get practical help from neighbors if you should need it?

**1**=very difficult **2**=difficult **3**=possible **4**=easy **5**=very easy
